# Short term outcomes of extremely low birth weight infants from a multicenter cohort study in Guangdong of China

**DOI:** 10.1038/s41598-022-14432-2

**Published:** 2022-07-01

**Authors:** Chun-Hong Jia, Zhou-Shan Feng, Xiao-Jun Lin, Qi-Liang Cui, Sha-Sha Han, Ya Jin, Guo-Sheng Liu, Chuan-Zhong Yang, Xiao-Tong Ye, Yi-Heng Dai, Wei-Yi Liang, Xiu-Zhen Ye, Jing Mo, Lu Ding, Ben-Qing Wu, Hong-Xiang Chen, Chi-Wang Li, Zhe Zhang, Xiao Rong, Wei-Min Huang, Wei Shen, Bing-Yan Yang, Jun-Feng Lv, Le-Ying Huo, Hui-Wen Huang, Hong-Ping Rao, Wen-Kang Yan, Yong Yang, Xue-Jun Ren, Dong Liu, Fang-Fang Wang, Shi-Guang Diao, Xiao-Yan Liu, Chu-Ming You, Qiong Meng, Bin Wang, Li-Juan Zhang, Yu-Ge Huang, Dang Ao, Wei-Zhong Li, Jie-Ling Chen, Yan-Ling Chen, Wei Li, Zhi-Feng Chen, Yue-Qin Ding, Xiao-Yu Li, Yue-Fang Huang, Ni-Yang Lin, Yang-Fan Cai, Zhong-He Wan, Yi Ban, Bo Bai, Guang-Hong Li, Yue-Xiu Yan, Fan Wu

**Affiliations:** 1 Department of Pediatrics, The Third Affiliated Hospital of Guangzhou Medical University, Guangzhou, 510150 Guangdong China; 2 Department of Neonatology, The First Affiliated Hospital of Jinan University, Guangzhou, 510630 Guangdong China; 3 Department of Neonatology, Shenzhen Maternal and Child Healthcare Hospital, Affiliated Southern Medical University, Shenzhen, 518028 Guangdong China; 4 Department of Neonatology, Foshan Maternal and Child’s Hospital, Foshan, 528000 Guangdong China; 5 Department of Neonatology, Women and Children Hospital of Guangdong Province, Guangzhou, 510010 Guangdong China; 6 Department of Neonatology, Shenzhen People’s Hospital, Shenzhen, 518020 Guangdong China; 7 Department of Neonatology, Meizhou People’s Hospital, Meizhou, 514031 Guangdong China; 8 Department of Neonatology, Guangzhou Women and Children’s Medical Center, Guangzhou, 510120 Guangdong China; 9 Department of Neonatology, Nanfang Hospital of Southern Medical University, Guangzhou, 510515 Guangdong China; 10 Department of Neonatology, Boai Hospital of Zhongshan, Zhongshan, 528400 Guangdong China; 11 Department of Neonatology, Zhuhai Maternity and Child Health Hospital, Zhuhai, 519001 Guangdong China; 12 Department of Neonatology, Huizhou Municipal Central Hospital, Huizhou, 516001 Guangdong China; 13 Department of Neonatology, Dongguan Maternity and Child Health Hospital, Dongguan, 523002 Guangdong China; 14 Department of Neonatology, Jiangmen Central Hospital, Affiliated Jiangmen Hospital of Sun Yat-Sen University, Jiangmen, 529000 Guangdong China; 15 Department of Neonatology, Yuebei People’s Hospital, Shaoguan, 512026 Guangdong China; 16 Department of Neonatology, Guangdong Second Provincial People’s Hospital, Guangzhou, 510317 Guangdong China; 17 Department of Pediatrics, Zhujiang Hospital of Southern Medical University, Guangzhou, 510280 Guangdong China; 18 Department of Pediatrics, The Affiliated Hospital of Guangdong Medical University, Zhanjiang, 524001 Guangdong China; 19 Department of Neonatology, The Second Affiliated Hospital of Shantou University Medical College, Shantou, 515041 Guangdong China; 20 Department of Neonatology, Jinan University Medical College Affiliated Dongguan Hospital, Dongguan, 523900 Guangdong China; 21 Department of Pediatrics, Dongguan People’s Hospital, Dongguan, 523000 Guangdong China; 22 Department of Neonatology, The First Affiliated Hospital of Sun Yat-Sen University, Guangzhou, 510080 Guangdong China; 23 Department of Neonatology, The First Affiliated Hospital of Shantou University Medical College, Shantou, 515041 Guangdong China; 24 Department of Neonatology, Nanhai District People’s Hospital of Foshan, Foshan, 528200 Guangdong China; 25 Department of Neonatology, Huadu District People’s Hospital of Guangzhou, Guangzhou, 510800 Guangdong China; 26 Department of Pediatrics, The First People’s Hospital of Zhaoqing, Zhaoqing, 526020 Guangdong China; 27 Guangdong Provincial Key Laboratory of Major Obstetric Diseases, Guangzhou, 510150 Guangdong China

**Keywords:** Paediatric research, Neonatology, Preterm birth

## Abstract

With the increase in extremely low birth weight (ELBW) infants, their outcome attracted worldwide attention. However, in China, the related studies are rare. The hospitalized records of ELBW infants discharged from twenty-six neonatal intensive care units in Guangdong Province of China during 2008–2017 were analyzed. A total of 2575 ELBW infants were enrolled and the overall survival rate was 55.11%. From 2008 to 2017, the number of ELBW infants increased rapidly from 91 to 466, and the survival rate improved steadily from 41.76% to 62.02%. Increased survival is closely related to birth weight (BW), regional economic development, and specialized hospital. The incidence of complications was neonatal respiratory distress syndrome (85.2%), oxygen dependency at 28 days (63.7%), retinopathy of prematurity (39.3%), intraventricular hemorrhage (29.4%), necrotizing enterocolitis (12.0%), and periventricular leukomalacia (8.0%). Among the 1156 nonsurvivors, 90.0% of infants died during the neonatal period (≤ 28 days). A total of 768 ELBW infants died after treatment withdrawal, for reasons of economic and/or poor outcome. The number of ELBW infants is increasing in Guangdong Province of China, and the overall survival rate is improving steadily.

## Introduction

Low birth weight preterm infants have a particularly high risk for morbidity and mortality^1,2^. In recent decades, the outcomes of preterm infants, especially extremely preterm (defined as gestational age [GA] < 28 weeks) and extremely low birth weight (ELBW, defined as birth weight [BW] < 1000 g) infants, have improved worldwide due to the use of antenatal steroids, pulmonary surfactant treatment and advances in perinatal health care, such as neonatal resuscitation, mechanical ventilation and nutritional management^3–4^. However, the mortality and morbidity vary widely across countries or regions. Generally, more improvements have been gained in developed countries or regions, such as the United States^2,5^, the United Kingdom^6^, Japan^7,8^ and Singapore^9^.

Available data of extremely preterm and ELBW infants are very important for family counseling and clinical practice improvement. Many neonatal networks or collaborative study groups, such as the Eunice Kennedy Shriver National Institute of Child Health and Human Development Neonatal Research Network (NICHD NRN) in the United States^5,10^, Canada Neonatal Network (CNN)^11^, Neonatal Research Network of Japan (NRNJ)^8,12^, Etude Epidémiologique sur les Petits Ages Gestationnels (EPIPAGE) in France^13^, and EPICure in the United Kingdom^6,14^, have worked well and continuously monitored the outcomes of these infants. However, in mainland China, a similar national or provincial network has not been established. The outcomes of extremely preterm and ELBW infants observed in a large population remain unclear. Therefore, we initiated a collaborative study group including twenty-six neonatal intensive care units (NICUs) from Guangdong Province of China to perform a multicenter survey of the short-term outcomes at discharge of extremely preterm or ELBW infants from 2008 to 2017. Generally, GA and BW are the two most important indicators of the maturity for preterm infants. In a previous paper, based on GA, the outcomes of extremely preterm infants were summarized and analyzed^15^. Similarly, based on BW, the outcomes of ELBW infants should be demonstrated. In addition, BW was recommended to group the preterm infants by the World Health Organization and had been used in many studies. This study can be helpful for making comparisons with other studies by BW categories and benefit for continuous quality improvement plan.

The outcomes of ELBW infants can be affected by many aspects such as maternal disorders, fetal or neonatal diseases, perinatal and neonatal care etc. The most influential factors for survival may be the equipment in the NICUs and the skills of the personnel who handle the neonates. In this study, we tried to summarize and analyze the demographics of ELBW infants and their mothers; the survival rate variation among discharged years, BW categories (per 100 g) and regions, or between types of discharged hospital; the major complications of BW categories (per 100 g); the survival days (or hours) of the non-survivors, and the causes for care withdrawal.

## Results

### Demographics of ELBW infants and mothers

**Figure 1:**
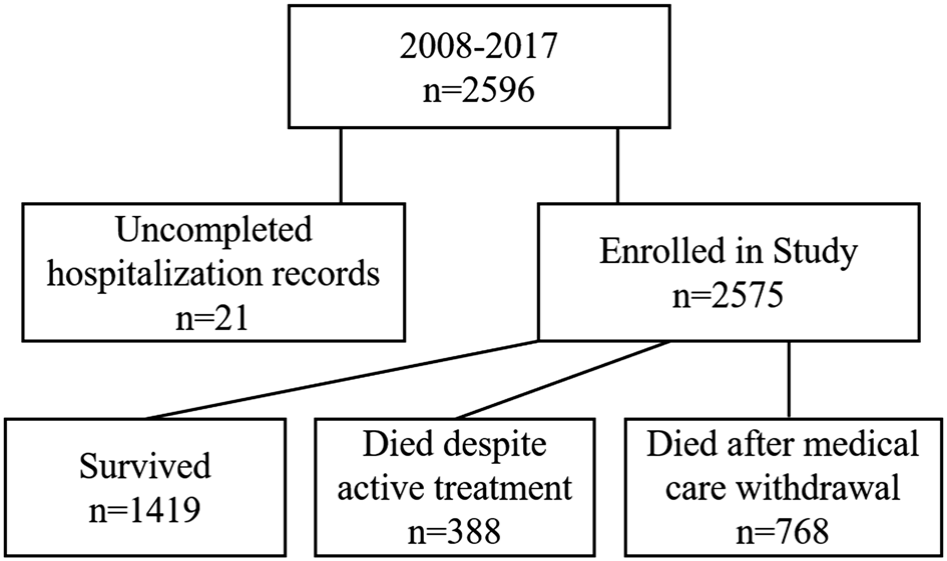
Extremely low birth weight infants discharged from 2008 to 2017 enrolled in the study.

From 2008 to 2017, there were 2596 ELBW infants discharged from the participating NICUs. As there were twenty-one infants excluded for uncompleted hospitalization records, the rest 2575 ELBW infants were enrolled in this survey (Fig. 1). The overall survival rate at discharge was 55.11% (1419 of 2575). In total, the median BW was 900 (800, 950) grams, and the distribution ranged from 22 (0.85%) for less than 500 g, 52 (2.02%) for 500–599 g, 150 (5.83%) for 600–699 g, 372 (14.45%) for 700–799 g, 685 (26.60%) for 800–899 g to 1294 (50.25%) for 900–999 g. The lowest BW in the survivors was 480 g. The mean GA was 27.96 ± 2.06 weeks, and the distribution ranged from 26 (1.01%) for less than 24 weeks, 102 (3.96%) for 24 weeks, 246 (9.55%) for 25 weeks, 420 (16.31%) for 26 weeks, 515 (20.00%) for 27 weeks, 521 (20.23%) for 28 weeks, 315 (12.23%) for 29 weeks to 430 (16.70%) for equal to or above 30 weeks.

To clarify the perinatal factors and outcome of ELBW infants, we specifically grouped the ELBW infants based on survival, shown in Table 1. Both the BW and GA in survivor group were greater than nonsurvivor group (*p* < 0.001). Comparing with the nonsurvivor group, there were fewer infants in the survivor group with Apgar scores ≤ 3 at 1 min and ≤ 3 or 4–7 at 5 min (all *p* < 0.001). The survivor group had a longer hospital stay and a higher rate of receiving surfactant therapy (*p* < 0.001), but there was no significant difference in two or more doses of surfactant therapy. Interestingly, increasing percentage of small for gestational age (SGA) infants was found in survivor group. No significant difference in sex existed between the two groups.

**Table 1:** Demographics of extremely low birth weight (ELBW) infants and the mothers in outcome categories.

Characteristics	Survivors (N = 1419)	Nonsurvivors (N = 1156)	OR (95% CI)	*p*-value
**Characteristics of infants**				
Gender (male), n (%)	745 (52.50)	623 (53.89)	0.946 (0.809–1.105)	NS
GA (weeks), mean ± SD	28.37 ± 1.95	27.45 ± 2.08	–	< 0.001
BW (grams), median (IQR)	910 (840, 960)	850 (750, 930)	–	< 0.001
SGA, n (%)	360 (25.37)	234 (20.24)	1.339 (1.111–1.614)	< 0.01
**Apgar score, n (%)**				
≤ 3 at 1 min	125 (8.81)	209 (18.08)	0.438 (0.345–0.555)	< 0.001
4–7 at 1 min	479 (33.76)	431 (37.28)	0.857 (0.729–1.008)	NS
≤ 3 at 5 min	14 (0.99)	48 (4.15)	0.230 (0.126–0.498)	< 0.001
4–7 at 5 min	148 (10.23)	251 (21.71)	0.420 (0.337–0.523)	< 0.001
Surfactant therapy (any dose), n (%)	1150 (81.04)	801 (69.29)	1.895 (1.579–2.274)	< 0.001
Surfactant therapy (two doses or more), n (%)	166 (11.70)	164 (14.19)	0.801 (0.636–1.010)	NS
Length of hospital stay (days), median (IQR)	69 (53, 85)	3 (1, 11)	–	< 0.001
**Characteristics of mothers**				
History of pregnancy problems^a^, n (%)	589 (41.51)	452 (39.10)	1.105 (0.943–1.295)	NS
Age ≥ 35 years, n (%)	310 (21.85)	221 (19.12)	1.183 (0.975–1.435)	NS
Cesarean section, n (%)	691 (48.70)	399 (34.52)	1.801 (1.535–2.113)	< 0.001
Twin/multiple pregnancy, n (%)	530 (37.35)	466 (40.31)	0.883 (0.753–1.035)	NS
Antenatal corticosteroid, n (%)	800 (56.38)	467 (40.40)	1.907 (1.629–2.232)	< 0.001
Premature rupture of membranes, n (%)	342 (24.10)	203 (17.56)	1.491 (1.228–1.810)	< 0.001
Infection in perinatal period, n (%)	78 (5.50)	62 (5.36)	1.026 (0.728–1.446)	NS
Gestational diabetes mellitus, n (%)	123 (8.67)	84 (7.27)	1.211 (0.907–1.617)	NS
Pregnancy induced hypertension syndrome, n (%)	398 (28.05)	240 (20.76)	1.488 (1.239–1.787)	< 0.001
Placental abruption/Placenta previa, n (%)	115 (8.10)	101 (8.74)	0.921 (0.697–1.218)	NS
Cervical incompetence, n (%)	20 (1.41)	35 (3.03)	0.458 (0.263–0.798)	< 0.01
Fetal distress, n (%)	108 (7.61)	64 (5.54)	1.406 (1.021–1.935)	< 0.05

GA, gestational age; BW, birth weight; SD, standard deviation; IQR, interquartile range; SGA, small for gestational age; OR, odds ratio; CI, confidence intervals; NS, no significant difference.

^a^History of pregnancy problems refers to that the mother had at least one of the histories as follow: spontaneous abortion, induced abortion, stillbirth, preterm birth, ectopic pregnancy, or baby died during neonatal period.

Comparing with the nonsurvivor group, the mothers in the survivor group had a higher proportion of antenatal steroid therapy and cesarean section (both *p* < 0.001), and a lower incidence of cervical incompetence (*p* < 0.01). Interestingly, the mothers in the survivor group had higher incidences of premature rupture of membranes (PROM) (*p* < 0.001), fetal distress (*p* < 0.05) and pregnancy-induced hypertension (PIH) syndrome (*p* < 0.001). Between the two groups, a similar prevalence was found in the history of pregnancy problems, mother’s age (≥ 35 years), multiple pregnancy (twins or triplets), infection in the perinatal period, gestational diabetes mellitus, and placental disease (placental abruption or placenta previa).

### Survival rates of ELBW infants improved with increasing BW

**Figure 2:**
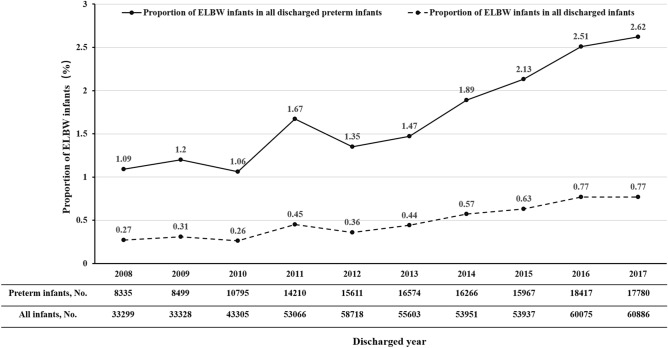
The proportion of ELBW infants in all discharged preterm infants or in all discharged infants from 2008 to 2017. The chi square test linear-by-linear association showed that the proportions of ELBW infants in all discharged preterm infants or in all discharged infants increased annually (both *P* < 0.001).

Both the number and survival rates of ELBW infants increased from 2008 to 2017. The number of ELBW infants discharged from the involved NICUs increased rapidly from 91 cases in 2008 to 466 cases in 2017 (Table 2). Moreover, the proportion of ELBW infants among all discharged preterm infants rose annually from 1.09% in 2008 to 2.62% in 2017 (*p* < 0.001), and the proportion of ELBW infants among all discharged infants increased annually from 0.27% in 2008 to 0.77% in 2017 (*p* < 0.001, Fig. 2). It was encouraging that the survival rate of ELBW infants improved steadily from 41.76% in 2008 to 62.02% in 2017 (*p* < 0.001, Table 2).

**Table 2:** The survival rate of extremely low birth weight (ELBW) infants at discharge from 2008 to 2017.

Discharged year	2008	2009	2010	2011	2012	2013	2014	2015	2016	2017	*p*-value
ELBW infants, n	91	102	114	237	210	244	308	340	463	466	–
BW (grams), median (IQR)	900 (800, 965)	895 (785, 945)	893 (800, 950)	900 (800, 945)	900 (824, 960)	917 (821, 965)	900 (800, 950)	900 (800, 959)	880 (790, 950)	870 (780, 940)	–
Survival, n (%)	38 (41.76)	48 (47.06)	56 (49.12)	110 (46.41)	110 (52.38)	136 (55.74)	164 (53.25)	195 (57.35)	273 (58.96)	289 (62.02)	< 0.001*
Died under active treatment, n (%)	13 (14.29)	14 (13.73)	17 (14.91)	39 (16.45)	23 (10.95)	27 (11.07)	57 (18.51)	61 (17.94)	77 (16.63)	60 (12.88)	–
Died after treatment withdrawal, n (%)	40 (43.96)	40 (39.22)	41 (35.96)	88 (37.13)	77 (36.67)	81 (33.20)	87 (28.25)	84 (24.71)	113 (24.41)	117 (25.11)	–

*Chi-square test linear-by-linear association. ELBW: Extremely low birth weight; IQR: Interquartile range.

There were twenty-two infants weighing less than 500 g at birth, and only one survived. With the increase per 100 g in BW between 500 and 999 g, the number of ELBW infants increased sharply from 52 in the group with BW 500–599 g to 1294 in the group with BW 900–999 g. In the same time, the survival rate rose dramatically from 30.77% in the group with BW 500–599 g to 65.53% in the group with BW 900–999 g (*p* < 0.001, Table 3).

**Table 3:** The survival rate of extremely low birth weight (ELBW) infants in relation to birth weight.

Birth weight (grams)	< 500	500–599	600–699	700–799	800–899	900–999	*p*-value
ELBW infants, n	22	52	150	372	685	1294	–
Survived, n (%)	1 (4.55)	16 (30.77)	46 (30.67)	145 (38.98)	363 (52.99)	848 (65.53)	< 0.001*
Died under active treatment, n (%)	2 (9.09)	15 (28.85)	43 (28.67)	63 (16.94)	110 (16.06)	155 (11.98)	–
Died after treatment withdrawal, n (%)	19 (86.36)	21 (40.38)	61 (40.67)	164 (44.09)	212 (30.95)	291 (22.49)	–

ELBW, extremely low birth weight.

*Chi-square test linear-by-linear association.

### Variations in survival rates among different regional economic development and hospital categories

According to the prosperity of regional economic development, the collaborative NICUs could be divided into three levels. Specifically, Guangzhou and Shenzhen (including eleven NICUs) belonged to the high level, the other cities in the Pearl Delta (including ten NICUs) belonged to the medium level, and the cities outside the Pearl Delta (including five NICUs) belonged to the low level. From the low level to the high level, the overall survival rates increased sharply (*p* < 0.05, Table 4). Among the twenty-six NICUs involved, seven were in specialist hospitals (maternal and children’s hospitals), and the others were in general hospitals. The overall survival rate of ELBW infants in specialist hospitals was higher than that in general hospitals (*p* < 0.001, Table 4). To further clarify the influencing cofactors to the survival rate of ELBW infants, binary logistic regression model was used. And it suggested that BW, GA, discharged year, regional economic development and hospital categories were associated with the survival rate of ELBW infant (Table 5).

**Table 4:** Differences of survival rate among regions or between hospitals.

	Regional economic development				Hospital categories		
	Low-level	Middle-level	High-level	*p*-value	Specialist hospitals	General hospitals	*p*-value
NICUs, n	5	10	11	–	7	19	–
ELBW infants, n	352	728	1495	–	1068	1507	–
BW (grams), median (IQR)	885 (800, 950)	900 (813, 960)	890 (790, 950)	–	880 (790, 950)	900 (800, 950	–
Survived, n (%)	175 (49.72)	400 (54.95)	844 (56.45)	< 0.05*	642 (60.11)	777 (51.56)	< 0.001^#^
Died under active treatment, n (%)	69 (19.60)	93 (12.77)	226 (15.12)	–	133 (12.45)	255 (16.92)	–
Died after treatment withdrawal, n (%)	108 (30.68)	235 (32.28)	425 (28.43)	–	293 (27.43)	475 (31.52)	–

NICUs, Neonatal intensive care units; ELBW, extremely low birth weight; IQR, interquartile range.

*Chi-square test linear-by-linear association.

^#^Pearson’s Chi-square test.

**Table 5:** Binary logistic regression analysis of influencing factors for survival rate.

Influencing factors	*β* value	Wald *χ*^2^	*p*	OR (95% CI)
Birth weight	0.004	105.750	< 0.001	1.004 (1.004–1.005)
Gestational age	0.190	65.632	< 0.001	1.209 (1.155–1.266)
Discharged year	0.095	31.151	< 0.001	1.100 (1.064–1.137)
Regional economic development	0.131	4.848	0.028	1.140 (1.015–1.281)
Type of hospital	0.486	29.262	< 0.001	1.626 (1.363–1.939)

### Complications of ELBW infants during hospitalization

The incidences of major complications in ELBW infants were 85.2% for neonatal respiratory distress syndrome (RDS), 63.7% for oxygen dependency at 28 days, 39.3% for any grade of retinopathy of prematurity (ROP), 29.4% for any grade of intraventricular hemorrhage (IVH), 12.0% for any stage of necrotizing enterocolitis (NEC) and 8.0% for periventricular leukomalacia (PVL). With the increase per 100 g in BW between 500 and 999 g, Chi-square test linear-by-linear association showed a significant decreasing trend in RDS, oxygen dependency at 28 days, ROP ≥ grade 3, IVH (any grade) and IVH ≥ grade III incidence, respectively (all *p* < 0.001); but this tendency was not found in incidence of ROP (any grade), NEC (any stage and stage ≥ IIb) or PVL (Table 6).

**Table 6:** The incidence of complications during hospitalization in ELBW infants.

	< 500 g (n = 22)	500–599 g (n = 52)	600–699 g (n = 150)	700–799 g (n = 372)	800–899 g (n = 685)	900–999 g (n = 1294)	Total (n = 2575)	*p*-value*
**RDS**								
Assessed, n	22	52	150	372	685	1294	2575	
Diagnosed, n (%)	21 (95.5)	46 (88.5)	141 (94.0)	331 (89.0)	579 (84.5)	1075 (83.1)	2193 (85.2)	< 0.001
**Oxygen dependency at 28 days**								
Assessed, n	5	18	58	140	360	821	1402	
Diagnosed, n (%)	5 (100.0)	16 (88.9)	46 (79.3)	111 (79.3)	249 (69.2)	466 (56.8)	893 (63.7)	< 0.001
**ROP**								
Assessed, n	5	22	71	187	412	819	1516	
Diagnosed (any grade), n (%)	1 (20.0)	10 (45.5)	32 (45.1)	77 (41.2)	155 (37.6)	321 (39.2)	596 (39.3)	NS
Diagnosed (≥ grade 3), n (%)	0	8 (36.4)	10 (14.1)	29 (15.5)	44 (10.7)	62 (7.6)	153 (10.1)	< 0.001
**NEC**								
Assessed, n	12	38	102	242	508	1074	1976	
Diagnosed (any stage), n (%)	0	6 (15.8)	19 (18.6)	32 (13.2)	52 (10.2)	128 (11.9)	237 (12.0)	NS
Diagnosed (≥ stage IIb), n (%)	0	1 (2.6)	5 (4.9)	5 (2.1)	15 (3.0)	21 (2.0)	47 (2.4)	NS
**IVH**								
Assessed, n	12	38	92	252	493	958	1845	
Diagnosed (any grade), n (%)	8 (66.7)	15 (39.5)	38 (41.3)	95 (37.7)	140 (28.4)	246 (25.7)	542 (29.4)	< 0.001
Diagnosed (≥ grade III), n (%)	6 (50.0)	6 (15.8)	16 (17.4)	32 (12.7)	33 (6.7)	51 (5.3)	144 (7.8)	< 0.001
**PVL**								
Assessed, n	12	38	92	252	493	958	1845	
Diagnosed, n (%)	0 (0)	1 (2.6)	5 (5.4)	20 (7.9)	45 (9.1)	77 (8.0)	148 (8.0)	NS

RDS, respiratory distress syndrome; ROP, retinopathy of prematurity; NEC, necrotizing enterocolitis; IVH, intraventricular hemorrhage; PVL, periventricular leukomalacia; NS, no significant difference.

*Chi-square test linear-by-linear association were used to compare the groups of 500–599 g, 600–699 g, 700–799 g, 800–899 g and 900–999 g.

### Survival time and the reasons for withdrawal treatment in nonsurvivors

Among the 1156 nonsurvivors, 90.0% (1040 of 1156) of infants died during the neonatal period (≤ 28 days), and the other 10.0% (116 of 1156) died after the neonatal period (> 28 days). Specifically, 27.2% (314 of 1156) of infants died during the first 24 h, 16.4% (190 of 1156) on the second day, 9.4% (109 of 1156) on the third day, 15.2% (176 of 1156) during the fourth to seven days, 9.7% (112 of 1156) in the second week, 7.4% (85 of 1156) in the third week and 4.7% (54 of 1156) in the fourth week. The survival time of nonsurvivors under active treatment or withdrawal treatment were shown in Table 7. The chi-square test showed that there was a significant difference in the distribution of survival days in nonsurvivors between active treatment and treatment withdrawal (*p* < 0.001).

**Table 7:** Survival time of the nonsurvivors.

Age of death	ENP				LNP			ANP
	≤ 24 h	~ 2 d	~ 3 d	~ 7 d	~ 14 d	~ 21 d	~ 28 d	> 28 d
Died under active treatment, n (%)	80 (20.6)	58 (14.9)	33 (8.5)	57 (14.7)	43 (11.1)	26 (6.7)	25 (6.4)	66 (17.0)
Died after treatment withdrawal, n (%)	234 (30.5)	132 (17.2)	76 (9.9)	119 (15.5)	69 (9.0)	59 (7.7)	29 (3.8)	50 (6.5)
Total, n (%)	314 (27.2)	190 (16.4)	109 (9.4)	176 (15.2)	112 (9.7)	85 (7.4)	54 (4.7)	116 (10.0)

ENP, early neonatal period; LNP, late neonatal period; ANP, after neonatal period.

In this study, 768 ELBW infants died after withdrawal treatment. The potential reasons were summarized and analyzed. For 35.9% (276 of 768) of infants, there were concerns about the economic burden together with the fear of poor or uncertain outcomes; for 29.6% (227 of 768) of infants, there were only fears of poor or uncertain outcomes; and for 14.7% (113 of 768) of infants, there were concerns only about the economic burden. Besides, for 1.6% (12 of 768) of infants, medical treatments were withdrawn owing to other factors, such as gender preference, and for 18.2% (140 of 768) of infants, the exact reasons were not mentioned.

## Discussion

The outcome of ELBW infants has gradually attracted worldwide attention in recent decades. Guangdong Province locates in southern China, with a population of more than 100 million and a highly developed economy and industrialization. In this study, we confirmed that the number of ELBW infants increased rapidly from 2008 to 2017 in Guangdong Province. More importantly, the survival rate improved steadily year by year. These data provide useful information to complement the understanding of ELBW infants in developing countries.

From the 1990s or 2000s, the number of ELBW infants began to increase in many developed countries^16–18^. Similar to the reports from other countries, our study also suggested a significant increase in ELBW infants over the ten years, from 1.09 per 1000 discharged infants in 2008 to 2.62 per 1000 discharged infants in 2017. A 2.4-fold increase was noted. Although this is not a national population-based survey, it can partly reflect the situation of ELBW infants in China.

During the past decades, the mortality rate of ELBW infants has decreased in many developed countries or regions. In Japan, the rate of mortality and the mortality in ELBW infants during NICU were 13.0% and 17.0% in 2005^7^. In the United States, the standardized mortality rates for infants weighing 501–750 g and 751–1000 g in 2009 were 36.6% and 11.7%, respectively^19^. In Korea, the survival rate of ELBW infants increased dramatically from 14.0% in 1985–1989 to 69.6% in 2010–2014^20^. However, in China, it was reported that just half of ELBW infants survived in 2011^21^. In our survey, the overall survival rate of ELBW infants at discharge was 55.11%. Encouraging improvement has been made, from 41.76% in 2008 to 62.02% in 2017.

Although the economy of Guangdong Province is relatively developed, the regional development is still unbalanced. In results of regional comparisons, we found that ELBW infants in economically developed regions had higher survival rates than those in less economically developing regions. This difference within Guangdong Province may be a microcosm in China. China is a multiprovincial country with unbalanced economic development. Hong Kong, a developed modern city neighboring Guangdong Province, reported higher survival rate^21^. In China, specialist hospitals, such as children’s hospitals or maternal and children’s hospitals, always have better facilities and more favorable policies in neonatal care than general hospitals. As a result, a higher survival rate was noted in the specialist hospitals in our study. A similar phenomenon was found in another multicenter study from China^21^.

Perinatal management is essential for the outcomes of ELBW infants. Many studies have shown that antenatal corticosteroids effectively decrease the mortality of preterm infants and even reduce various complications, such as RDS, NEC, IVH and ROP^22,23^. Although there is still some controversy regarding the side effects^24^, there is a consensus that the advantages of prenatal corticosteroids outweigh the disadvantages^25,26^. Unfortunately, only 49.2% of ELBW infants’ mothers received antenatal corticosteroids in our study, but it was 80%-90% in developed countries^10,27,28^. Therefore, this situation urgently needs to change.

Interestingly, numerous studies have shown that PROM and PIH syndrome are high-risk factors for preterm delivery and infant death^29,30^, but our study showed that the incidence of PROM and PIH syndrome in the survivor group was higher than that in the nonsurvivor group. Moreover, cesarean section was more common in the survivor group. A possible explanation is that PROM or PIH syndrome could have been an early warning that attracted the attention of pregnant women and led them go to the hospital for help in time. When they were admitted to the hospital, more active medical care, such as antenatal corticosteroids, cesarean section, neonatal resuscitation and pulmonary surfactant, was given. Nevertheless, the other potential reasons still need to be further studied and analyzed.

ELBW infants are unstable and tend to suffer various complications due to their prematurity. Without active life support, many infants die during the neonatal period, especially in the first 7 days of age, and some die due to critical illnesses despite receiving active treatments. In our study, 90.0% of nonsurviving infants died during the neonatal period, while nearly 68.3% died in the first 7 days, and the majority died after withdrawal treatment (Table 6). Although withdrawal treatment in these infants is a controversial issue, it truly exists in developing countries because of the high hospital costs as well as the high risk for poor outcome^31^. We can reasonably believe that the survival of ELBW infants will continue to improve with the economic development and medicine advancement in China.

To the best of our knowledge, this study covers the largest population sample and the longest time span involved in studies of ELBW infants in China to date. It provides useful information for family consultation, clinical practice and further research. However, there are obvious limitations in this study. It is not a population-based or nationwide study. Moreover, the long-term outcomes of ELBW infants are not addressed, and further studies are needed.

## Conclusion

In conclusion, this survey presents an overall short-term outcome of ELBW infants in Guangdong Province of China. Both the number and the survival rate of ELBW infants increased annually from 2008 to 2017.

## Methods

### Participating hospitals and study population

The Collaborative Study Group for Extremely Preterm and Extremely Low Birth Weight Infants was founded with an aim to investigate the prevalence and the short-term outcomes of extremely preterm and ELBW infants in Guangdong Province, China. To ensure the survey can be performed feasibly and representatively, the enrolled hospitals were strictly selected as the neonatology department must be the Clinical Key Specialty of Guangdong Province or the representative of medical units offering neonatal intensive care in their respective areas. Even more, the regional distribution of these hospitals was also considered. At last, twenty-six hospitals were involved. The Third Affiliated Hospital of Guangzhou Medical University was responsible for coordinating this study.

The ELBW infants discharged from the NICUs of the collaborative hospitals for the first time between Jan 1st, 2008 and Dec 31st, 2017 were eligible for this study. Only the infants with uncompleted hospitalization records were excluded. The unstable infants transferred to other hospitals or discharged after treatment withdrawal were received follow-up visit in out-patient or by telephone during the study.

All methods were carried out in accordance with relevant guidelines and regulations. The study protocol was approved by the Institutional Review Board of the Third Affiliated Hospital of Guangzhou Medical University and by the Ethics Committees of the Third Affiliated Hospital of Guangzhou Medical University. Written informed consent was obtained from the parents at the time of admission.

### Data collection

The study protocol was fully discussed by all members, and a standardized questionnaire for data collection, including maternal and neonatal demographics, treatments and major complications during hospitalization, and outcomes at discharge was designed. The same diagnostic criteria were applied to all enrolled NICUs. The relevant records of all enrolled infants and their mothers were reviewed thoroughly, and a questionnaire was completed carefully. All sheets were sent to the Third Affiliated Hospital of Guangzhou Medical University, and the data from each questionnaire were input into the database. To minimize bias among centers and investigators, comprehensive and systematic training was provided to the staff involved in the survey. The data collected by the researchers at each collaborative NICU were supervised and checked by the director of the NICU, who was responsible for quality assurance. The records were also checked for accuracy and completeness by collaborative centers.

### Definitions and classifications

In this survey, surviving infants were defined as neonates who survived to the time of discharge. GA was calculated from the date of the last menstrual period or was determined by fetal ultrasound assessment. SGA was defined as newborns whose birth weight is lower than the 10th percentile of birth weight in infants of the same gender and gestational age. RDS was diagnosed in preterm infants with the onset of respiratory distress shortly after birth and a compatible chest radiograph appearance^32,33^. The criteria utilized in our survey for the diagnosis of NEC and for grading the severity of disease were based on Bell’s stage^34^. ROP and the graded standard were defined by the international classification of ROP^35^. IVH and PVL were diagnosed by cranial ultrasonography or magnetic resonance imaging (MRI). The Papile grading system was used to grade IVH^36^, and PVL was defined as degeneration of white matter adjacent to the cerebral ventricles following cerebral hypoxia or brain ischemia^37^. Due to the definition of BPD remained inconsistent, we directly descripted and calculated the infants with “oxygen dependency at 28 days”.

### Statistical analysis

All statistical analyses were performed using SPSS 18.0 for Windows (IBM, Armonk, NY, USA). Continuous variables were presented as the mean ± standard deviation (*SD*) or as median and interquartile range (*IQR*) according to the distributions. Categorical variables were presented as counts and percentages. To compare the variation between two groups, *t-tests* or *Mann–Whitney tests* were used in continuous variables; while *Pearson’s Chi‑square test* was used in categorical variables and presented with odds ratio (OR) and 95% confidence intervals (CI). In addition, *Chi-square tests linear-by-linear association* were used to compare the survival rates among discharged years, BW categories (per 100 g), regions and the major complication among BW categories (per 100 g), respectively. To further clarify the influencing cofactors (BW, GA, discharged year [1 = 2008, 2 = 2009, 3 = 2010, 4 = 2011, 5 = 2012, 6 = 2013, 7 = 2014, 8 = 2015, 9 = 2016, 10 = 2017], regions of economic development level [1 = low-level, 2 = middle-level, 3 = high-level] and type of hospital [1 = general hospitals, 2 = specialist hospitals]) to the outcome (0 = nonsurvivor, 1 = survivor) of the ELBW infants, binary logistic regression was used. The test level was set at *α* = 0.05, and the cutoff of *p* < 0.05 was considered statistically significant.

### Ethics declarations

Data collection was approved by the Institutional Review Board of the Third Affiliated Hospital of Guangzhou Medical University. Written informed consent was obtained from the parents at the time of admission.

## Data Availability

The datasets used and analyzed during the current study are available from the corresponding author on reasonable request.
